# Subtelomeric FISH analysis in 76 patients with syndromic developmental delay/intellectual disability

**DOI:** 10.1186/1824-7288-35-9

**Published:** 2009-04-27

**Authors:** Elga F Belligni, Elisa Biamino, Cristina Molinatto, Jole Messa, Mauro Pierluigi, Francesca Faravelli, Orsetta Zuffardi, Giovanni B Ferrero, Margherita Cirillo Silengo

**Affiliations:** 1 Dipartimento di Scienze Pediatriche, University of Torino, Torino, Italy; 2 Dipartimento di Biologia Generale e Genetica Medica, University of Pavia, Pavia, Italy; 3 S.C. Genetica Umana, E.O.Ospedali Galliera, Genova, Italy

## Abstract

**Background:**

Intellectual disability affects approximately 1 to 3% of the general population. The etiology is still poorly understood and it is estimated that one-half of the cases are due to genetic factors. Cryptic subtelomeric aberrations have been found in roughly 5 to 7% of all cases.

**Methods:**

We performed a subtelomeric FISH analysis on 76 unrelated children with normal standard karyotype ascertained by developmental delay or intellectual disability, associated with congenital malformations, and/or facial dysmorphisms.

**Results:**

Ten cryptic chromosomal anomalies have been identified in the whole cohort (13,16%), 8 in the group of patients characterized by developmental delay or intellectual disability associated with congenital malformations and facial dysmorphisms, 2 in patients with developmental delay or intellectual disability and facial dysmorphisms only.

**Conclusion:**

We demonstrate that a careful clinical examination is a very useful tool for pre-selection of patients for genomic analysis, clearly enhancing the chromosomal anomaly detection rate. Clinical features of most of these patients are consistent with the corresponding emerging chromosome phenotypes, pointing out these new clinical syndromes associated with specific genomic imbalances.

## Background

Developmental delay (DD) and intellectual disability (ID) represent common conditions affecting 1 to 3% of the general population and it has been estimated that one-half of the cases are due to genetic factors [1-4]. With a prevalence of 5 to 16% and 1 to 2% of cases respectively, trisomy 21 and Fragile X syndrome are the most common genetic causes of ID [5].

Routine cytogenetic analysis detects chromosomal aberrations involving at least 3–5 megabases (Mb) of DNA, in concordance with the technique resolution power. Fluorescent in situ hybridisation (FISH) overcomes this limitation allowing to investigate specific loci or subtelomeric regions for cryptic aberrations that are responsible of roughly 5 to 7% of all DD/ID cases [6,7]. These anomalies represent one of the most common causes of idiopathic DD/ID [8-11], being identified in 6.3% to 10.2% of moderate to severe DD/ID and in a significantly lower rate, less than 1%, in mildly retarded patients [12]. Major malformations and/or dysmorphisms, pre and post-natal growth retardation, and/or positive family history can be observed in the majority of patients with moderate to severe DD/ID related to criptic chromosomal imbalances [9,13-15]. As a matter of fact, clinical pre-selection of DD/ID patients improves the detection rate. It has been suggested for this purpose the use of specific check-list, such as the five item of De Vries et al. [16]. In recent years the study of genotype-phenotype correlations of these anomalies has allowed the definition of new emerging chromosomal phenotypes [15,17,18]. The recent introduction of the technology of array comparative genomic hybridization (CGH), that allows the detection of submicroscopic copy number variations in the whole genome, represents the next step forward in this effort.

We performed subtelomeric FISH analysis in 76 unrelated children affected by various degree of DD/ID, congenital malformations (CM) and facial dysmorphisms (FD), with normal standard karyotype. Ten subtelomeric anomalies have been identified (13.16%), underlying the role of cryptic subtelomeric anomalies in the pathogenesis of complex clinical presentation associated with DD/ID.

## Methods

### Patients

Seventy-six patients, aged from 3 days to 14 years, recruited at the Department of Pediatrics, University of Torino, were enrolled in the study. In order to better define the clinical features correlated with chromosomal subtelomeric imbalances, we have divided the cohort in three subgroups: 32/76 patients (42,1%) with DD/ID associated with CM and striking FD, 18/76 patients (23,68%) with DD/ID associated with FD, 26/76 patients (34,21%) with DD/ID associated with CM and not relevant FD.

### Methods

Routine cytogenetic analysis at 400–550 bands level was performed in all patients and it did not detect any imbalance. Chromosome preparations from peripheral blood cells were used for FISH analysis. The Chromoprobe-T kit with telomeric specific clones was used according to the supplier's instructions (Cytocell, UK) with minor modifications. When a criptic subtelomeric rearrangements was identified by FISH, prometaphase chromosomes were re-analysed in order to rule -out if the rearrangement could have been detected in retrospect.

## Results

**Figure 1:**
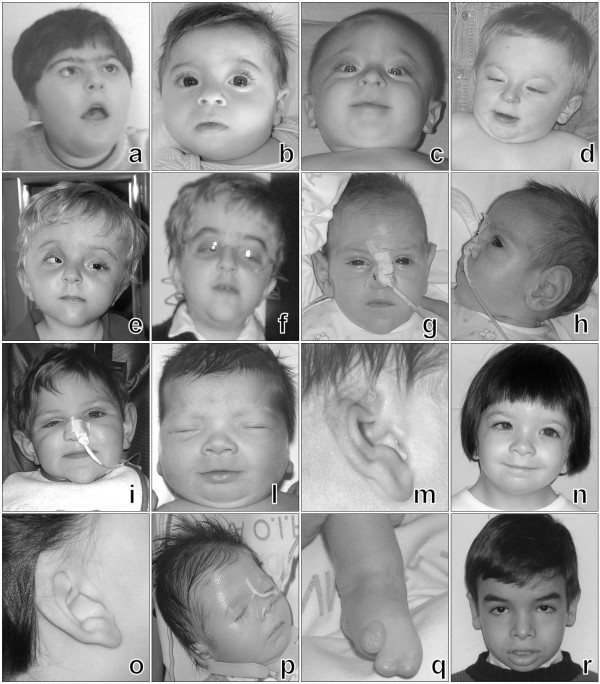
**Facial aspects and specific malformations in patients with criptic subtelomeric anomalies**. (a) Patient 3: note round face, low frontal hairline, hypertelorism and synophris; (b) Patient 4: note epicanthal folds, upslanting palpebral fissures and low-set ears; (c-d) Patient 5: note mild trigonochephaly and evolution of the phenotype at follow-up; (e-f) Patient 6: note plagiochephaly, palpebral ptosis and evolution of the phenotype at follow- up; (g-h-i) Patient 7: note macrotia and evolution of the phenotype at follow-up; (l-m-n-o) Patient 8: note severe hypertelorism, epicanthal folds and right external ear malformation and their evolution at follow-up; (p-q) Patient 9: note flat face, low-set ears and foot malformation; (r) Patient 10: note the coarse face.

Ten cryptic chromosomal anomalies have been identified in the cohort (13,16%), 3 *de novo *deletions (2 patients with 1p del and 1 patient with 9q del), 4 unbalanced translocations of parental origin (1 patient with der(9)t(9;16)(9pter-9q34.3::16q24.3–16qter)pat; 1 patient with der(20)t(16;20) (q24;q13.3)pat; 1 patient with der(6)t(6;1)(p22.3;q44)mat and 1 patient with der(7)t(7;12)(q34;q24.32)mat), and 3 *de novo *unbalanced translocations (1 patient with der(6)(ptel-, qtel++); 1 patient with der(5) t(5;10)(pter;qter); 1 patient with t(1;13)(p32.2;q31.1). In particular 8/10 anomalies have been identified in the first group, namely DD/ID associated with CM and FD, with a group-specific detection rate of 25%; 2/10 anomalies have been identified in the second group, namely DD/ID associated with FD, with a group-specific detection rate of 11.11%; in the third group, namely DD/ID associated with CM, no subtelomeric aberrations were identified. [Table 1 and Fig 1].

**Table 1:** Clinical presentation of patients affected by subtelomeric anomalies.

Case	Gender Age At The Diagnosis	Subtelomeric Anomaly	DD/ID Relevant neurologic impairment At diagnosis	Evolution of the neurologic phenotype At follow-up	Facial Dysmorphisms	Congenital Malformations	Growth	
							
							Prenatal	Postnatal
1	Female 4 y.o.	Deletion 1p36 46, XX, ish del 1p36 de novo	DD Partial seizures EEG: focal irritative complexes	Lost at follow-up	Frontal bossing, convergent strabismus, straight eyebrows, deep set eyes, low-set ears	none	Normal	Normal

2	Female 16 m.o.	Deletion 1p36 46, XX, ish del 1p36 de novo	DD – Nystagmus Generalized seizures EEG: right temporal spike and wave activity, multifocal temporal irritative complexes	Lost at follow-up	Straight eyebrows, deep set eyes, divergent strabismus	VSD and bicuspid aortic valve	Normal	Normal

3	Male 8 y.o.	Deletion 9q34 46, XY, ish del(9)(q34.3qter) de novo	Severe ID	Lost at follow up	Round face, low frontal hairline, hypertelorism, synophris.	Corpus callosum dysgenesis, stenosis of pulmonary valve, ASD, genitalia hypoplasia	Normal	Obesity

4	Male 7 m.o.	Trisomy 16q24.3, Monosomy 9q34 46, XY, ish der(9)t (9;16)(9pter-9q34.3::16q24.3-16qter) pat	DD	3 y.o: severe DD, microcephaly	Hypertelorism, upslanting palpebral fissures, epicanthal folds, low-set ears, large and indented tip of the nose	Anterior diaphragmatic hernia Morgagni type, agenesis of corpus callosum, multiple skeletal anomalies	IUGR	Normal

5	Male 7 m.o.	Trisomy 16q24, Monosomy 20q13.3 46, XY, ish der (20) t(16;20)(q24;q13.3) pat	DD	6 y.o: severe DD, aggressive and food seeking behaviour, microcephaly,	Hypertelorism, ptosis, upslanting palpebral fissures, strabismus, long philtrum, thin upper lip	Trigonocephaly, neonatal arthrogryposis, penis recurvation, severe hypospadias, bilateral cryptorchidism recurrent urinary tract infections.	Normal	Obesity

6	Male 1 y.o.	Trisomy 6p22.3, Monosoy 1q44 46, XY, ish der(6) t(6;1)(p22.3;q44)mat	DD	8 y.o: normal motor development and borderline cognitive development	Bilateral ptosis, micrognathia, hypoplastic teeth, low-set ears	Complex craniosynostosis, hypoplastic corpus callosum, renal hypoplasia, progressive renal failure leading to renal transplantation	IUGR	Normal

7	Male 3 d.o.	Trisomy 12q 24.32, Monosomy 7q 34 46, XY, ish der(7) t(7;12)(q34;q24.32) mat	Hypotonia	2 y. o.: severe DD, microcephaly	macrotia	Caudal regression, chorioretinal coloboma, VSD, intestinal malrotation with diaphragmatic hernia, hypospadia, bilateral cryptorchidism, skeletal abnormalities, congenital bilateral hip dislocation and congenital luxation of right rotula, clubfeet	IUGR	Microsomia

8	Female 2 d.o.	Trisomy 6q, Monosomy 6p 46, XX, ish der(6)(ptel-;qtel++) de novo	None	7 m.o: mild motor delay 5 y.o: normal motor and cognitive development	Hypertelorism, epicanthal folds, short neck with pterigium	External ear malformation, posterior embriotoxon.	Normal	Normal

9	Female 6 d.o.	Trisomy 10q, Monosomy 5p 46, XX, ish der(5)t(5pter;10qter) de novo	Severe hypotonia	2 y.o.: severe DD, microcephaly 3 y.o: severe DD, microcephaly	Flat facies, downslanting palpebral fissures, low-set ears	ASD, VSD, II- III-IV toes syndactyly, high-arched palate	IUGR	Normal

10	Male 7 y.o.	Trisomy 1p32.2, Monosomy 13q31.1 46, XY, ish t(1;13)(p32.2;q31.1) de novo	Mild ID, macrocephaly	Lost at follow up	Prominent forehead, deep set eyes, thick superior lip, prominent inferior lip	None	Normal	Normal

### Patient 1

A 4 year-old girl was evaluated for DD and seizures. She was born at 37 weeks of gestation, after an uneventful dizygotic pregnancy, with a birth weight of 2.45 kg (3^rd ^percentile), length 48 cm (10^th ^percentile), head circumference 32 cm (3^rd ^percentile). APGAR scores were 7 and 8 at the first and fifth minute respectively. DD was evident in the first two years of life: she sat alone at 13 months and she stood at 27 months. Partial seizures with focal irritative complexes at EEG were diagnosed at 12 months of age. When she was evaluated frontal bossing, convergent strabismus, straight eyebrows, deep-set eyes and low-set ears were noted. She was not able to walk and language was limited to few words. Subtelomeric FISH analysis detected a *de novo *terminal deletion of chromosome 1p (46,XX.ish.del (1p)(pVYS218C-)).

### Patient 2

A 16 month-old girl was evaluated for DD and seizures. She was born at 37 weeks of gestation by spontaneous delivery after an uneventful pregnancy. Neonatal weight was 2.49 kg (25^th ^percentile), length 47.8 cm (25^th ^percentile), head circumference 32.2 cm (3^rd ^percentile). APGAR scores were 9 at the first and fifth minute. At birth, a ventricular septal defect (VSD) and a bicuspid aortic valve were detected by ecochardiogram. She was able to sit alone at 7 months, but indipendent walking was still not achieved at 16 months. At 10 months she presented generalized seizures characterized by gaze, circumoral cyanosis, masticatory movements. Right temporal spike and wave activity, associated with irritative multifocal anomalies were recorded on EEG; even if valproic acid therapy was subsequently undertaken, EEG anomalies persisted for 1 year and the children experienced febrile seizures during the second year of life. Moreover, external hydrocephalus was detected by MRI. On physical examination at 16 months, weight was 10.3 kg (10^th^–25^th ^percentile), head circumference 47 cm (50^th ^percentile), length 80 cm (50^th ^percentile); straight eyebrows, deep set eyes and divergent strabismus were noted. Ophtalmological examination revealed bilateral nystagmus and hypermetropic astigmatism. Subtelomeric FISH analysis revealed a *de novo *terminal deletion of chromosome 1p (46, XX, ish.del (1p)(pVYS218C-)).

### Patient 3

A 8 year-old boy was referred for severe ID and CM. He was born at 38 weeks of gestation by non consanguineous parents after a pregnancy complicated by echocardiographic detection of a pulmonary valve stenosis in the third trimester. Birth weight was 2.32 kg (3^rd ^percentile), no other neonatal parameters were reported. At birth, the stenosis of the pulmonary valve was confirmed, and an associated atrial septal defect (ASD) was observed. Corpus callosum dysgenesis was detected by cerebral ultrasound and CT scan and later confirmed by cerebral MRI. When he was evaluated, his weight was 28 kg (75^th ^percentile), head circumference 49 cm (25^th ^– 50^th ^percentile), length 123 cm (25^th ^– 50^th ^percentile). He was not able to sit nor to speak. Facial phenotype was characterized by round face, low frontal hairline, hypertelorism, synophris. Genitalia were hypoplastic and a mild obesity was evident. Subtelomeric FISH analysis revealed a de novo 9q34-qter deletion (46,XY,ish. del (9q)(q34.3qter)).

### Patient 4

A 7 month-old boy was evaluated for sever DD and CM. He was born at 37 weeks of gestation by caesarean section due to intra-uterine growth restriction (IUGR) and pre-natal diagnosis of corpus callosum agenesis, confirmed in the neonatal period by cerebral MRI. Birth weight was 2.34 kg (3^rd ^percentile), length 48 cm (50^th ^percentile), head circumference 33.5 cm (25^th ^percentile). APGAR scores were 8 and 9 at the first and fifth minute. At 7 months an anterior diaphragmatic hernia, Morgagni type, and multiple skeletal anomalies were revealed (enlarged sternal heads of both clavicles, costal broadening and flaring, ovoid hypoplasia of L1 and L2 vertebral bodies, large iliac wings and short metacarpi), associated with hypertelorism, upslanting palpebral fissures, epicanthal folds, low-set ears, large and indented tip of the nose and severe DD. Follow-up at 3 years of age revealed a weight of 15 kg (75^th ^percentile), height 94 cm (50^th ^percentile) and OFC 45.5 cm (< 3^rd ^percentile); neurodevelopment milestones were severely delayed, with no functional speech and absence of independent walking. Association of corpus callosum agenesis with diaphragmatic hernia initially suggested the diagnosis of Donnai-Barrow syndrome [19]. Subtelomeric FISH analysis showed a paternally derived translocation consisting of partial 1.2 Mb deletion of 9q and partial 2.7 Mb trisomy of 16q (46, XY, ish der(9)t(9;16)(9pter-9q34.3::16q24.3-16qter)).

### Patient 5

A 7 month-old boy was evaluated for DD and CM. He was born at 37 weeks of gestation, after a pregnancy complicated by oligohydramnios. Birth weight was 2.48 kg (3^rd ^percentile), length and head circumference were not reported. On neonatal physical examination, trigonocephaly, mild distal arthrogryposis, hypospadia, penis recurvation, bilateral cryptorchidism were noted and a grade III vescico-ureteral reflux was detected. On physical examination at 7 months his weight was 7 kg (3^th ^percentile), length was 64 cm (< 3^rd ^percentile), head circumference 41.5 cm (3^rd ^percentile). DD was evident and associated with hypertelorism, upslanting palpebral fissures and convergent strabismus. The follow-up at 6 years of age revealed a severe ID, associated with an aggressive and food seeking behaviour, with language limited to 3 words. Head circumference was 48 cm (< 3^rd ^percentile) and weight was 27 kg (> 97^th ^percentile), height not reported. Facial phenotype was characterized by ptosis, long philtrum and thin upper lip. Subtelomeric FISH analysis revealed a paternally derived translocation, consisting with partial deletion of chromosome 20q and partial duplication of chromosome 16q (46, XY, ish der (20)t(16;20)(q24;q13.3) pat).

### Patient 6

A 1 year-old boy was referred for DD and CM. Family history revealed a maternal uncle affected by ID and progressive chronic renal failure who died at 16 years of age for acute pneumonia. The child was born by spontaneous delivery at 36 weeks of gestation after a pregnancy complicated by IUGR, oligohydramnios, mild hypertension and gestational diabetes. Birth weight was 2.13 kg (< 3^rd ^percentile), head circumference 30 cm (3^rd ^percentile), length not reported. He presented a complex cranial vault anomaly, characterized by brachicephaly and anterior plagiochephaly, associated with bilateral renal hypoplasia. When he was evaluated at 12 months of age, weight was 5 kg (<< 3^rd ^percentile), length 56 cm (<< 3^rd ^percentile), head circumference 44 cm (3^rd ^percentile); bilateral ptosis, micrognathia with hypoplastic teeth and low-set ears were evident. Cerebral MRI revealed a hypoplastic corpus callosum. Surgical correction of cranial vault anomaly was performed, with good aesthetic and neurologic outcome. At 3 years of age, he developed chronic renal failure; kidney biopsy was consistent with membranous glomerulonephritis and he underwent kidney transplantation at the age of 4. At follow up at 4 years, weight was 9.5 kg (<< 3^rd ^percentile), length 85 cm (<< 3^rd ^percentile). When he was revaluated at 8 years of age, a good recovery of auxometric parameters was evident, being his weight 25 kg (50^th ^percentile) and his height 121 cm (50^th ^percentile). Moreover he was attending a normal school program, with a back-up teacher. Subtelomeric FISH analysis revealed an unbalanced maternally derived translocation with a partial trisomy of chromosome 6p and partial monosomy of chromosome 1q (46, XY, ish der(6) t(6;1)(p22.3;q44)mat). The balanced chromosomal anomaly identified in the mother was also present in a phenotypically normal maternal uncle.

### Patient 7

A 3 day-old boy was evaluated for hypotonia, CM and FD. He was born at 36 weeks of gestation after a pregnancy complicated by IUGR. A previous miscarriage was reported. Birth weight was 1.8 kg (< 3^rd ^percentile), length and head circumference were not reported. At birth, facial dysmorphisms, microcephaly, macrotia, hypospadia, bilateral cryptorchidism, anal atresia and club feet were noted. Chorioretinal coloboma, neurological bladder, VSD, intestinal malrotation with right diaphragmatic hernia complicated the phenotype. Multiple skeletal abnormalities including fusion of the 10^th ^and 11^th ^ribs, sacral agenesis, congenital bilateral hip dislocation and luxation of the right rotula were observed at X-ray. At follow-up at 2 years of age severe DD was evident, being the child not able to sit nor to speak. Weight was 9.5 kg (< 3^rd ^percentile), length 70 cm (<< 3^rd ^percentile), head circumference 42 cm (<< 3^rd ^percentile). Subtelomeric FISH analysis revealed a maternally derived unbalanced translocation, resulting in a partial deletion of chromosome 7q and partial duplication of chromosome 12q (46, XY. ish der(7) t(7;12)(q34;q24.32) mat).

### Patient 8

A 7 month-old girl was evaluated for CM and FD at birth. Ultrasound detection of cystic hygroma in the first trimester led to perform karyotype on chorionic villi, resulting in a normal female karyotype. She was born at term, with a birth weight of 3.17 kg (25^th ^percentile), length 48 cm (25^th ^percentile), head circumference 33.5 cm (25^th ^percentile). APGAR scores were 8 and 9 at the first and fifth minute, respectively. At birth, right microtia, hypertelorism, epichantal folds, micrognathia and pterigium colli were noted. Acoustic oto-emission screening test was normal. Posterior embriotoxon was observed on ophtalmological examination. At 7 months of age, weight was 6.8 kg (3^rd ^percentile), length 64 (25^th ^percentile), head circumference 42 cm (25^th ^percentile). Mild motor DD associated with persistent neonatal phenotype, suggested the subtelomeric analysis, which revealed a *de novo*, chromosomal anomaly consistent with a partial monosomy of the short arm of chromosome 6 and a partial duplication of the long arm of the same chromosome (46, XX, ish der(6)(ptel-;qtel++) de novo). The long term follow-up at 5 years revealed a normal cognitive and motor development and auxometric parameters within normal limits (50^th ^percentile).

### Patient 9

A six day-old female was evaluated for hypotonia, CM and FD. Family history was remarkable for a first trimester spontaneous miscarriage. She was born at 41 weeks of gestation by caesarean section after a pregnancy complicated by IUGR. Birth weight was 2.2 kg (< 3^rd ^percentile), length 46.5 cm (3^rd ^percentile) and head circumference 31.5 cm (< 3^rd ^percentile); APGAR scores were 6 and 8 at the first and fifth minute, respectively. Severe hypotonia, flat facies, down-slanting palpebral fissures, high-arched palate, low-set ears and II, III, IV toes syndactyly were observed, associated with ASD and VSD. Tracheostomy was placed in the first month of life to prevent recurrent apnea and severe desaturation, and a feeding tube was positioned up to the first year of life. Cerebral MRI performed at 1 year of age revealed fronto-temporal cerebral atrophy. Follow-up at 2 years of life revealed severe DD, characterized by absent speech, poor head control and ineffective deglutition reflex. At 3 years of age, head circumference was 44.5 cm (<< 3^rd ^percentile), length 95 cm (50^th ^percentile), weight 11 kg (3^rd ^percentile). Language and independent walking were absent. Subtelomeric FISH analysis revealed a *de novo *unbalanced translocation with partial distal monosomy of chromosome 5p and partial distal trisomy of chromosome 10q (46, XX, ish der(5)t(5pter;10qter) de novo).

### Patient 10

A 7 year-old boy was evaluated for mild ID and FD. Family history was unremarkable for ID and CM. He was born at term after an uneventful pregnancy, with birth weight of 3.8 kg (75^th ^percentile), length 51 cm (50^th ^percentile), head circumference 36.5 cm (97^th ^percentile). APGAR scores were 9 and 10 at the first and fifth minute, respectively. Independent walking was reached at 24 months of age and language was completely absent at the age of 36 months. When he was evaluated at 7 years of age, a mild cognitive impairment was observed. Weight was 21 kg (50^th ^percentile), length 114 cm (50^th ^percentile) and head circumference 55 cm (> 97^th ^percentile). Facial dysmorphisms including prominent forehead, deep-set eyes, thick superior lip and prominent inferior lip first suggested the clinical diagnosis of α-talassemia mental retardation X linked syndrome (ATRX-syndrome; OMIM Number 30032). Cerebral MRI, EEG, visual evoked potentials (VEP), electroretinogram (ERG) and metabolic work-up were normal. Subtelomeric FISH analysis revealed a de novo translocation, consisting with a partial monosomy of 13q and partial duplication of 1p (46, XY, ish t(1;13)(p32.2;q31.1) de novo).

## Discussion

Chromosomal subtelomeric anomalies are a relevant cause of DD/ID and birth defects [14,20,21]. Here we present the results of a subtelomeric analysis performed on 76 paediatric patients, aged from 3 days to 14 years, presenting with a complex clinical phenotype associated with DD/ID. Ten cryptic chromosomal anomalies have been identified in the cohort, with a detection rate of 13.16%: 3 *de novo *deletions, 4 unbalanced translocations of parental origin, and 3 *de novo *unbalanced translocations. Interestingly we have observed relevant differences in the detection rate among the 3 groups identified on clinical features. In particular, the detection rate was 25% in the first group of patients (DD/ID, CM and FD), percentage reduced to 11,11% in the second one (DD/ID and FD), while no anomalies were identified in the third group (DD/ID and CM). This gradient in the detection rate reflects the probability that a chromosomal imbalance is responsible for complex developmental disturbances and leads to relevant facial dysmorphisms.

Some frequent cryptic telomeric anomalies are characterized by a specific phenotype and are emerging as recognizable subtelomeric syndromes, such as 1p terminal deletion (Patients 1–2). The phenotype is characterized by neurodevelopmental disability and a recognizable pattern of malformation associated with specific facial dysmorphisms, straight eyebrows, deep set eyes and frontal bossing that may direct towards the correct clinical diagnosis [10,17,18,22,23]. Also terminal 9q deletion (Patient 3) is a new emerging recognizable phenotype, described in about 30 patients [24], being characterized by severe ID, hypotonia, brachycephaly or microcephaly, flat face with hypertelorism, synophrys, anteverted nares, a distinctive mouth with macroglossia and a thickened lower lip, and congenital hearth defects. Moreover, sleep disturbances, autistic features and obesity due to food seeking behaviour have been reported in those patients [24-26]. Agenesis of corpus callosum associated with diaphragmatic hernia characterized Patient 4, suggesting the diagnosis of Donnai-Barrow syndrome (OMIM 222448). Subsequently an unbalanced t(9;16)(9qter;16q24.3qter)pat with partial monosomy 9q34-qter and partial trisomy 16q24-qter has been identified suggesting the hypothesis that 9q terminal deletion can cause Donnai-Barrow syndrome [19]. Patient 5 carried an unbalanced paternally derived der(20) t(16;20)(q24;q13.3), with partial 16q trisomy and 20q monosomy. Trisomy 16q24 is a clinical recognizable phenotype characterized by periorbital oedema in the neonatal period, generalized hypotonia, failure to thrive, severe DD/ID and a distinctive *facies *with a high forehead and bitemporal narrowing, associated with a variable genital and anal abnormalities [27,28]. This patient presented basopenienal hypospadia, supporting the hypothesis that one or more genes involved in uro-genital development are located in this chromosomal region [29,30]. Deletion of 20q13 is a very rare anomaly and it is associated with severe malformations of the limbs, short neck, flat occiput, midfacial dysmorphism, and failure to thrive [31-33]. Patient 6 carried a maternally derived unbalanced translocation, consisting with 6p trisomy and 1q monosomy. He presented a complex craniosynostosis, renal hypoplasia, hystologically characterized by membranous glomerulonephritis, leading to progressive renal failure. Interestingly, Pierpoint *et al *described a patient carrying a 6p terminal trisomy, who presented a striking overlapping facial phenotype associated with progressive renal failure [34]. These observations allow the hypothesis that a gene locus for syndromic renal failure maps to 6p22.3-pter. Moreover, we propose 6p terminal deletion as a new chromosomal syndrome, characterized by a peculiar facial appearance, renal failure and DD/ID. Interestingly the patient we described reached a good cognitive development and school performance after the surgical correction of the craniosynostosis, the kidney transplantation and an intensive rehabilitation program. Chromosome 1q terminal deletion [16,35-37] is emerging as a new specific chromosomal phenotype characterized by DD/ID, pre-postnatal growth retardation, microcephaly, seizures, bow shaped eyebrows, hand – foot anomalies and midline defects, including corpus callosum hypoplasia [36,38], and may probably contribute to the pathogenesis of the complex phenotype of this patient. The clinical presentation of Patient 7 confirms the role of genes mapping to terminal region of 7q in caudal regression pathogenesis [39]. Moreover, the peculiar facial traits, such as microcephaly, hypertelorism, epicanthus, coloboma/cataract, blepharoptosis/phimosis, broad nasal bridge with bulbous nasal tip, ear anomalies, short neck, and abnormal genitalia could be considered a specific phenotype for 7q34-qter deletion, as argued by Lukusa *et al*. [40]. Partial terminal 12q duplication is a rare event described in about 35 cases with mild phenotypical effects [41,42]. In Patient 8 facial dysmorphisms, characterized by hypertelorism and a striking external ear malformation, short neck associated with early motor developmental delay led to the clinical suspicion of 6p terminal deletion subsequently confirmed by subtelomeric FISH analysis. She is now 6 years old and she displays normal cognitive development at periodic clinical evaluation. This patient confirms that criptic chromosomal aberrations do not always result in DD/ID, suggesting caution in the definition of prognosis, particularly in prenatal genetic counselling. More extensive reports about the size of subtelomeric imbalances in normally neurodeveloped patients are needed in order to investigate which subtelomeric loci are implicated in syndromic DD/ID [43]. In Patient 9, IUGR, severe hypotonia associated with flat facies, hypertelorism, down-slanting palpebral fissures, and toes syndactyly first suggested us the clinical hypothesis of Smith-Lemli-Opitz syndrome (OMIM 270400). Subtelomeric FISH analysis revealed a partial monosomy 5p associated with trisomy 10q, already described in a single patient with an overlapping phenotype, characterized by hands and feet anomalies and facial dysmorphisms [44]. Patient 10 carried a partial monosomy 13q31.1 and partial trisomy 1p32.2. Eight patients with pure monosomy 13q33 have been previously reported, delineating a well-recognized syndrome, characterized by growth and psychomotor retardation, microcephaly and deficiency of coagulation factors VII and X [44,45]. Partial duplication of chromosome 1p has been described in 10 patients: major characteristics are abnormal genitalia in males, congenital heart defects, craniofacial and hand anomalies [46].

## Conclusion

Our report illustrates the relevance of careful dysmorphologic evaluation of patients with DD/ID variably associated with CM and/or FD as an useful tool for pre-selection for genomic analysis. This is particularly noteworthy in respect of the recent availability in the clinical setting of new technologies, such as array genomic hybridization, that allow whole-genome screening for sub-microscopic genomic anomalies, not previously detectable by conventional methods. Literature reports of patients affected by subtelomeric imbalances are very helpful in increasing clinicians awareness of subtle chromosomal phenotypes, and in revealing genomic candidate region for specific developmental defect. Paediatricians and clinical geneticists might improve their expertise in correlating new specific patterns of congenital anomalies with specific chromosomal imbalances, in order to optimize, both in terms of time and cost, the yield of chromosomal studies.

## Competing interests

The authors declare that they have no competing interests.

## Authors' contributions

BEF had made substantial contributions to acquisition of data and their interpretation, and had been actively involved in drafting the manuscript. BE had been involved in critical revision of data and helped to draft the manuscript. MC had been involved in acquisition of data and in their interpretation. MJ and PM carried out the molecular genetic studies. FF and ZO carried out the molecular genetic studies and revised them critically. FGB had been involved in drafting the manuscript and revised it critically. SCM conceived of the study, partecipated in its design, revised and critically interpretated data, coordinated and helped to draft the manuscript and critically revised it for important intellectual content. All authors read and approved the final manuscript.
